# Two-dimensional Talbot effect of the optical vortices and their spatial evolution

**DOI:** 10.1038/s41598-020-77418-y

**Published:** 2020-11-20

**Authors:** Denis A. Ikonnikov, Sergey A. Myslivets, Mikhail N. Volochaev, Vasily G. Arkhipkin, Andrey M. Vyunishev

**Affiliations:** 1 Kirensky Institute of Physics, Federal Research Center KSC SB RAS, Krasnoyarsk, Russia 660036; 2 Institute of Engineering Physics and Radio Electronics, Siberian Federal University, Krasnoyarsk, Russia 660041

**Keywords:** Imaging and sensing, Optics and photonics, Micro-optics

## Abstract

We report on the experimental and theoretical study of the near-field diffraction of optical vortices (OVs) at a two-dimensional diffraction grating. The Talbot effect for the optical vortices in the visible range is experimentally observed and the respective Talbot carpets for the optical vortices are experimentally obtained for the first time. It is shown that the spatial configuration of the light field behind the grating represents a complex three-dimensional lattice of beamlet-like optical vortices. A unit cell of the OV lattice is reconstructed using the experimental data and the spatial evolution of the beamlet intensity and phase singularities of the optical vortices is demonstrated. In addition, the *self-healing* effect for the optical vortices, which consists in flattening of the central dip in the annular intensity distribution, i.e., restoring the image of the object plane predicted earlier is observed. The calculated results agree well with the experimental ones. The results obtained can be used to create and optimize the 3D OV lattices for a wide range of application areas.

## Introduction

Structured light is a key issue of photonics, whose potential is coming to be implemented^1^. Usually, structured light represents the nontrivial intensity, polarization, and phase distributions of the coherent light, which can result in extraordinary characteristics capable of transforming the light–matter interactions. One of the brightest manifestations of structured light is the beams containing phase singularities also called *optical vortices*(OVs)^2^. Their feature is the orbital angular momentum (OAM) arising from the helical wavefront imposed by an azimuthal phase dependence of $\exp {(il\varphi )}$^3–5^, where *l* is the topological charge (TC) of the phase singularity^6^. The orbital angular momentum of a light beam can be transferred to matter^7^ and vice versa. Since the date of their discovery^2,3^, the OAM-carrying beams have found wide application in optical manipulations^8–11^, imaging^12,13^, and quantum key distribution^14^ and made it possible to implement the Tbit ${\hbox {s}}^{-1}$ data transmission rates in optical communications^15–18^. The recent progress in this field and future directions were reviewed in^5,19^. Among these directions are complex two- and three-dimensional (2D, 3D) lattices of optical vortices^20–22^. The formation of well-ordered spatial arrays of optical vortices is highly attractive, e.g., for micromanipulation in biology, medicine, and materials science^23^, two-dimensional optical trapping (creating arrays of microscale sculpted atom optical states in two and three dimensions)^1^ and sorting^24^, photolithography^25,26^, micromachining and structuring, etc.^27^ The most obvious approach to creating a spatial array of optical vortices is the use of division of a wavefront of the OAM-carrying beams. In this connection, it is reasonable to examine the near-field diffraction of the OAM-carrying beams at a 2D amplitude diffraction grating. It should be noted that the diffraction of the OAM-carrying beams was studied for the cases of a single slit^28,29^, sector-shaped diaphragm^30^, and 1D gratings^31^. It is well-known that a plane wave incident onto a periodic grating is diffracted such that the Talbot effect occurs in the near-field^32^. The physics behind this phenomenon is interference of diffracted waves, which results in the intensity distribution (image) self-reproduction at distances multiple of Talbot length $Z_T = 2 \Lambda ^2 / \lambda$, where $\Lambda$ is the grating period, $\lambda$ is the wavelength of the incident wave. This phenomenon is well-studied, both theoretically and experimentally, for plane waves^33^ and theoretically investigated for the beams comprising phase singularities^20,34,35^. In the experiment, the Talbot effect of the OAM-carrying beams was observed in the 1D case^31^, as well as for the two-dimensional configuration at the THz radiation^35,36^ and near-infrared single photons^37^. In the 1D case^31^, the experimental intensity patterns were in the form of a periodic set of stripes, while for two-dimensional configuration there is an array of beams with a topological charge. The Talbot effect was explored using a superposition of two optical lattices generated by a superposition of two quasi-OAM states in^38^ and exploited for generating optical vortex arrays by multiplexing metasurface design^22^. Till now, no consistent theoretical and experimental study of near-field diffraction of the optical vortices at a 2D amplitude diffraction grating in the visible range has been considered. Here, we report the results of observation of the two-dimensional Talbot effect of the optical vortices in the visible range and formation of three-dimensional lattices of optical vortices consisting of beamlet arrays and demonstrate their spatial evolution.

## Experimental and numerical results

**Figure 1:**
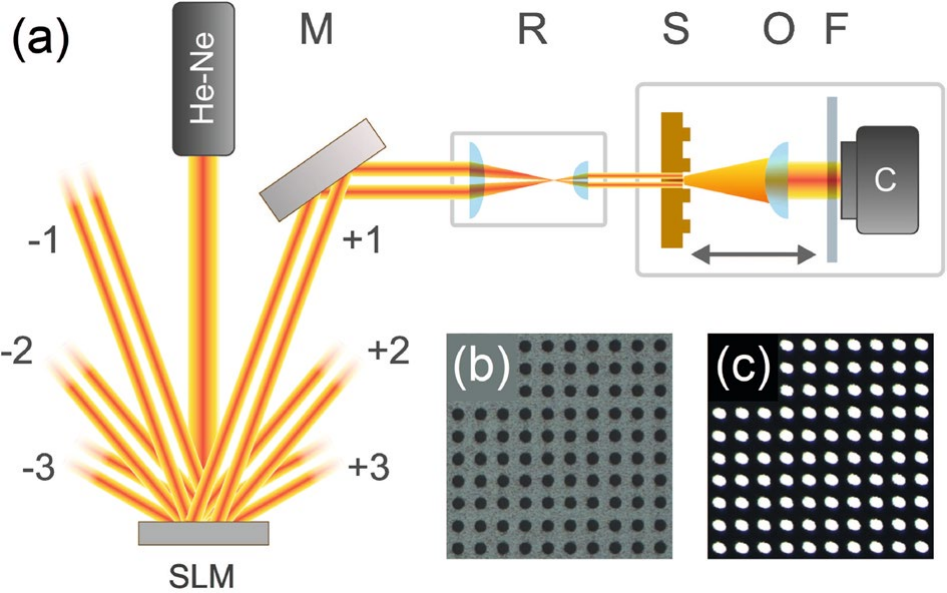
(**a**) Experimental setup. Spatial light modulator SLM, mirror M, reducer R, sample S, objective O, optical filter F, and CCD camera C. Optical images of a part of the 2D grating in (**b**) the reflection and (**c**) transmission mode.

**Figure 2:**
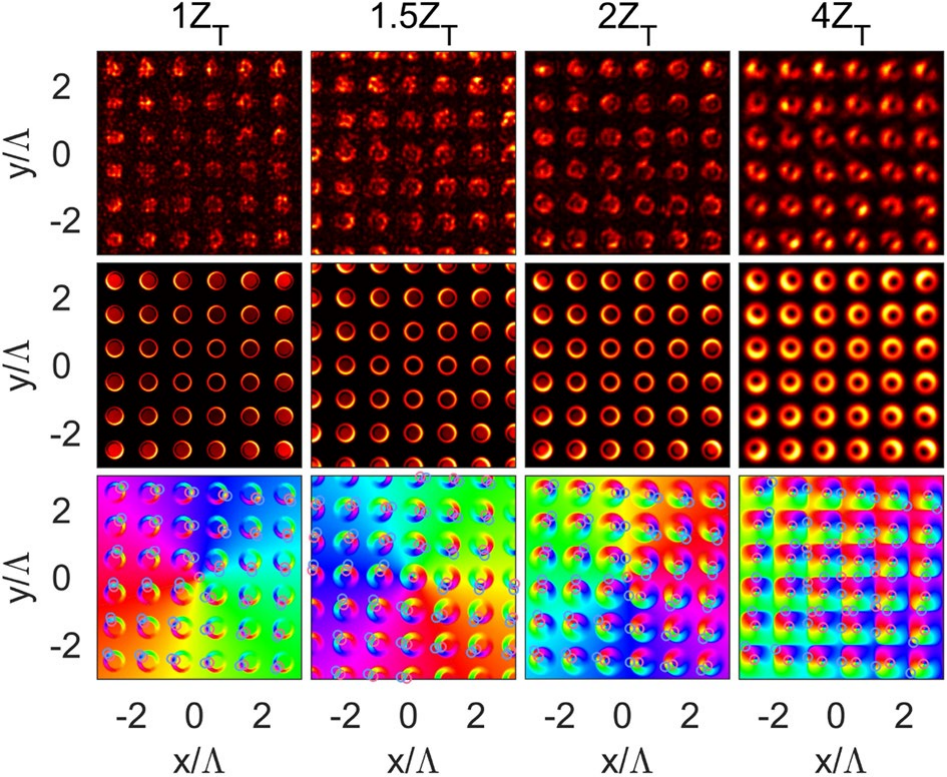
Experimental patterns (top row) and calculated intensity (middle row) and phase (bottom row) profiles in specific Talbot planes (columns) for the TC $l = +1$. Red and blue circles correspond to the positive and negative topological charges, respectively.

In the paper we study a near-field diffraction of the optical vortices at a 2D amplitude diffraction grating. In the experiments, the azimuthal phase modulation was imposed onto a Gaussian beam from a He–Ne laser using a 2D phase-only spatial light modulator (SLM, PLUTO-NIR-011, Holoeye, a pixel pitch of $8 \,\upmu \hbox {m}$). The laser source operated in a single-mode regime and produced the linearly polarized radiation at a wavelength of 632.8 nm. The laser radiation was expanded by a $3 \times$ beam expander before launching onto the SLM. A fork-shaped hologram loaded in the SLM provided the phase modulation in the form $\cos (G_{x} x + l\varphi )$, rather than $\exp (i\varphi )$, where $G_x$ is the reciprocal lattice vector. This allowed us to obtain the high-quality optical vortex in the first diffraction order, as shown in Fig. 1a. Then, the topological charge of the resulting vortex was measured by the method described in^39^. The beam with a specific TC was reduced and directed along the sample normal to the input facet. The amplitude 2D diffraction grating was located at the opposite side of the sample (the output facet) (See Methods for details concerning with the sample fabrication and characterization). Near-field diffraction patterns were imaged by a $100\times$ objective and measured using a monochrome CCD camera mounted on a motorized translation stage. As can be seen in Fig. 2 (top row), the measured intensity distributions (profiles) for the TC $l = +1$ consist of well-ordered 2D annular beamlet arrays with the ring-shaped maxima for all the Talbot planes, which differs from 1D case, where linear array of stripes is observed^31^. These maxima suffer from distortions caused by the weak intensity at the center of the incident annular beam. The shape of the maxima is different for each unit cells in the beamlet array. The unit cells are defined so that each one contains a single beamlet at its center and it approximately corresponds to a hole in the grating structure. The beamlets undergo deformations, which strengthen with distance from the center of the image plane and take a semicycle (U-shaped) form, as can be clearly seen for the forth Talbot plane. The beamlet positions coincide with the lattice points of the object structure for the integer Talbot planes, while the images corresponding to the fractional $m Z_T/2$ planes (see, for instance, the $3/2 Z_T$ plane) are shifted by half a period relative to those corresponding to the integer planes $Z_T$. Although this property is the same as in the classical Talbot effect, the annular beamlet arrays are observed instead of the hole images of the object structure. All the calculated results were obtained using the numerical simulation based on the Fresnel–Kirchhoff formalism described in Sec. Methods. The calculated intensity ($I(x,y,z) \sim |E(x,y,z)|^2$) and phase ($\psi (x,y,z) = \arg (E(x,y,z))$) profiles obtained using Eq. (13) are in good agreement with the experimental ones (Fig. 2). In the calculation, we used a beam radius of $w = 150~\upmu \hbox {m}$ in Eq. (2) to fit the intensity distributions to the experimental ones. Analysis of the phase distributions showed phase singularities in each beamlet array cell (Fig. 2, bottom row), which are distinguished by the vortex and anti-vortex, depending on the sign acquired. Here, we use the term *anti-vortex* for the vortex of the opposite sign. The phase increases counter-clockwise in the vicinity of the optical vortices with a positive TC. According to^5^, the TC of the optical vortex can be found using the path integral over the closed curve *C* subtended by a phase surface (over the field cross section) containing the singularities

1 $$\begin{aligned} TC = \frac{1}{2 \pi } \oint _{C} d \varphi \frac{\partial }{\partial \varphi } \psi (\mathbf{r },\varphi ). \end{aligned}$$

Here, $\psi (\mathbf{r },\varphi )$ is the phase distribution of a scalar wavefield presented in the polar coordinates. Eq. (1) allows us to calculate the TC of all optical vortices in the field cross section taking into account their values and signs, while their positions are defined by intersections of zero-level contours of both real and imaginary parts of the field. Although the optical singularities within each beamlet array cell are grouped in the vortex–anti-vortex pairs, the total TC over a single cell calculated using Eq. (1) is zero. This is not surprising and follows from the deterministic rule also known as the sign principle^40^ or *nucleation* of optical vortices based on the properties of the wavefields topology. In addition, there exists an uncompensated optical vortex at the center of the image (the so-called global vortex), which is related to the TC of the incident beam. The beamlets become broader and smoother with an increase in the propagation coordinate *z* or the number of a Talbot plane. They fill the entire cell area in the fourth Talbot plane and then begin overlapping. This trend is observed for the beamlet phase profiles as well.

**Figure 3:**
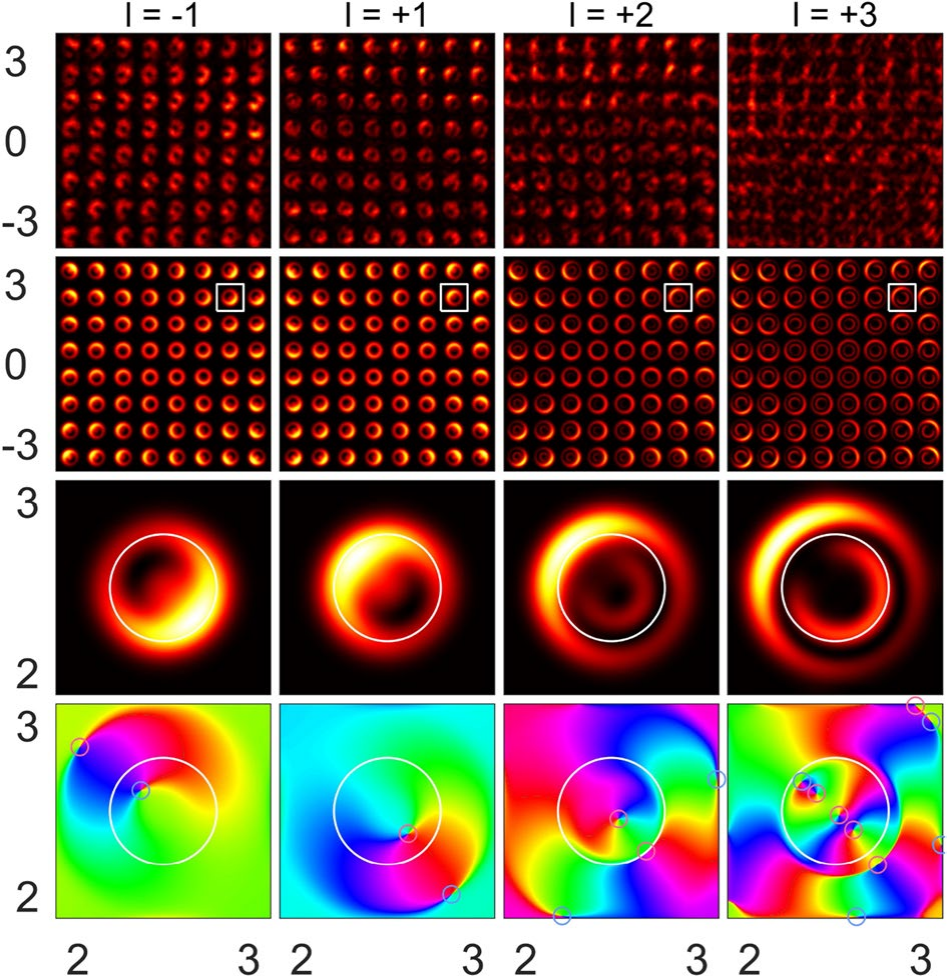
Experimental (top row) and calculated (the second row from the top one) intensity profiles for $8 \times 8$ periods and respective calculated intensity (the third row from the top one) and phase (bottom row) profiles for a single period (white squares in the previous row) for specific TCs of the incident beam ($l = -1, +1, +2$, and $+3$ in columns, from the left to the right) taken in the third Talbot plane. Red and blue circles correspond to the positive and negative topological charges, respectively.

**Figure 4:**
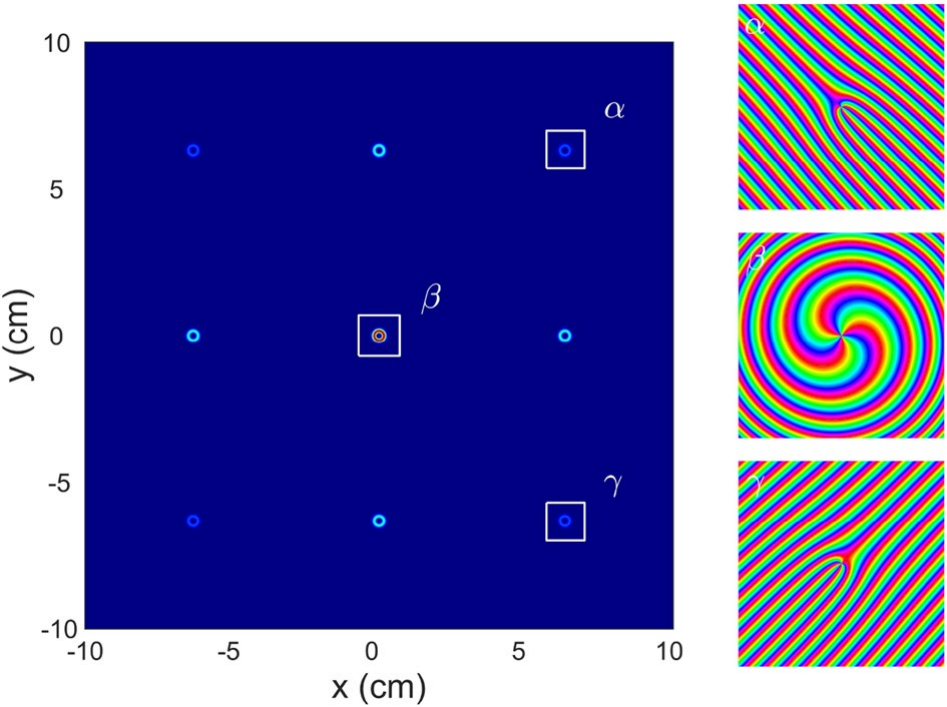
Calculated far-field diffraction pattern (left plot) at the distance 1 m and phase profiles for specific diffraction orders (right column) shown by squares in the left plot ($l = +3$).

Similar to the case of $l = +1$, the respective experiments were carried out for the incident beams with TC values of $l = -4, -3, -2, -1, +2,$ and $+3$. As was observed in the experiments and proved by our calculations, the beamlet arrays of the optical vortices were observed in all the cases. Figure 3 illustrates the results for TCs of $l = -1, +1, +2,$ and $+3$ at the third Talbot plane. All the distributions exhibit the rotational symmetry and have a four-fold symmetry axis. The change in the TC sign leads to the inversion of the observed images, as shown in Fig. 3(the first and second columns) for $l = -1$ and $l = +1$. When the TC increases, the light field configuration becomes complicated. For instance, at $l = +3$, the unit cell profile of a beamlet array consists of the double U-shaped maxima; one of them is embedded into the other and they are opposite to each other. Again, the field cross section contains off-axis phase singularities (optical vortices), which are predicted to be bounded by the vortex–anti-vortex pairs. The vortices–anti-vortices are connected by the curve pairwise, where the wavefront acquires a phase increment of $2 \pi$. This circumstance results in zero TC within each unit cell of the image. Despite this fact, the resulting TCs of the optical vortices within the area corresponding to round holes are equal to the TCs of the incident beam. In most cases, these vortices are spatially separated by signs, depending on the TC of the incident beam. The number and sign of the single optical vortices comprising inside the area corresponding to a grating hole are determined by the TC of the incident beam. However, this behavior is appropriate for a low (lower than 3) TC and a small (smaller than 4) number of the Talbot plane. In other cases, the number and sign of the optical vortices can change. For example, as shown in Fig. 3(right column), the phase patterns within the area corresponding to a single grating hole contain three vortices with $l = +1$ and one anti-vortex ($l = -1$), so the resulting TC is $+2$, while the TC of the incident beam is $l = +3$. It is clarified by Eq. (1) that all the phase singularities (over the incident beam cross section) retain the total TC of the incident beam, while the light field propagates through the entire grating. In other words, the global optical vortex associated with the TC of the incident beam remains in the position corresponding to the center of the structure in a given Talbot plane. An increase in the TC results in spreading of the beamlets and their overlap. The latter factor will result in the interaction between the beamlets. This complicates the intensity profiles of the beamlet array. It is supposed that the optical singularities will penetrate to the neighboring beamlet array cells, so they cannot be related to the given cell any more. In spite of such a behavior, the well-ordered diffraction pattern is formed in the far-field, as shown in Fig. 4. Individual diffraction orders represent the optical vortices whose TC is equal to the TC of the incident beam; in particular, in this case, we have $l = +3$. All the diffractive orders have fork-shaped phase profiles, except for the zero order, which has a spiral (azimuthal) phase profile.

**Figure 5:**
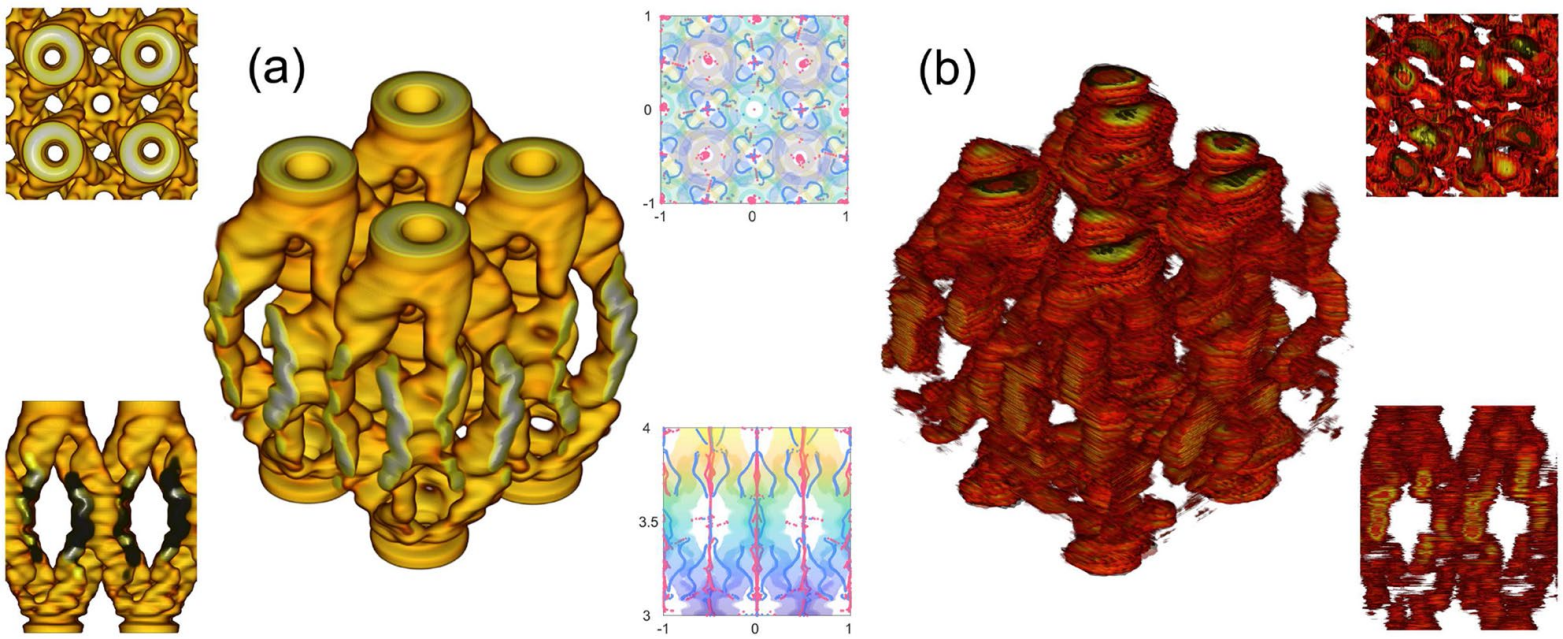
(**a**) Calculated beamlet isointensity volume and its top (top inset) and side (bottom inset) views (on the left). The insets on the right from the beamlets volume show the OV spatial evolution in the transverse (*xy*) plane (on the top) and horizontal (*xz*) plane (in the bottom). Spatial transformations of the OVs with the positive and negative signs are traced by the red and blue curves, respectively. (**b**) Experimental beamlets isointensity volume and its top (top inset) and side (bottom inset) views (on the right).

The results described above are related to the special case of diffraction at the integer and fractional Talbot planes. Of special interest is the analysis of the 3D spatial structure of the beamlets, i.e., the configuration of the optical vortices inside the area between two neighboring integer Talbot planes. Therefore, we numerically analyzed the spatial distribution of the intensity and phase singularities of the OVs. Figure5a shows the calculated beamlet isointensity volumes between the third and fourth Talbot planes for the $2\times 2$ central unit cell array for $l = +1$ and the respective top and side views (insets on the left). As can be seen, the beamlets undergo spatial evolution; specifically, each annular intensity beamlet is separated into four channels according to the number of neighboring lattice sites and then these channels merge with the formation of the annular intensity structures at the $3\frac{1}{2}$ Talbot plane placed between the initial beamlet positions in the *xy* plane corresponding to the third Talbot plane. After that, the annular structure is reconfigured back into the four channels and each of them contributes to the neighboring annular intensity maxima at the four Talbot planes. The spatial intensity profile of the beamlets at the fourth Talbot plane is sufficiently close to the profile corresponding to the third Talbot plane. Such a spatial behavior is typical of a rectangular lattice inside the area enclosed by any neighboring Talbot planes. To build an experimental beamlet structure, all the transverse profiles taken along the *z* axis were stacked in a single array. Then, the experimental beamlet structures were reconstructed using standard isovolume visualization tools. Figure5b shows the reconstructed experimental beamlets isointensity volume taken at the 1/*e* intensity level. Good agreement between the theoretical and experimental results proves our analytical model. The numerical analysis allows one to visualize the trajectories of OVs, which are shown in Fig. 5a (insets on the right). All optical vortices can be distinguished by the sign of the phase singularity and by its spatial behavior. The optical vortices with the positive TC nutate in the vicinity of array lattice points along the *z* axis, while the position of the global OV at the center of the lattice remains constant. The optical vortices with the negative TC have helical trajectories with the rotation axis coinciding with the intermediate position between the lattice points. These OVs are created in pairs and located opposite to each other relative to the rotation axis. The total number of positive OVs exceeds the number of negative OVs by a value of the TC of the incident beam.

**Figure 6:**
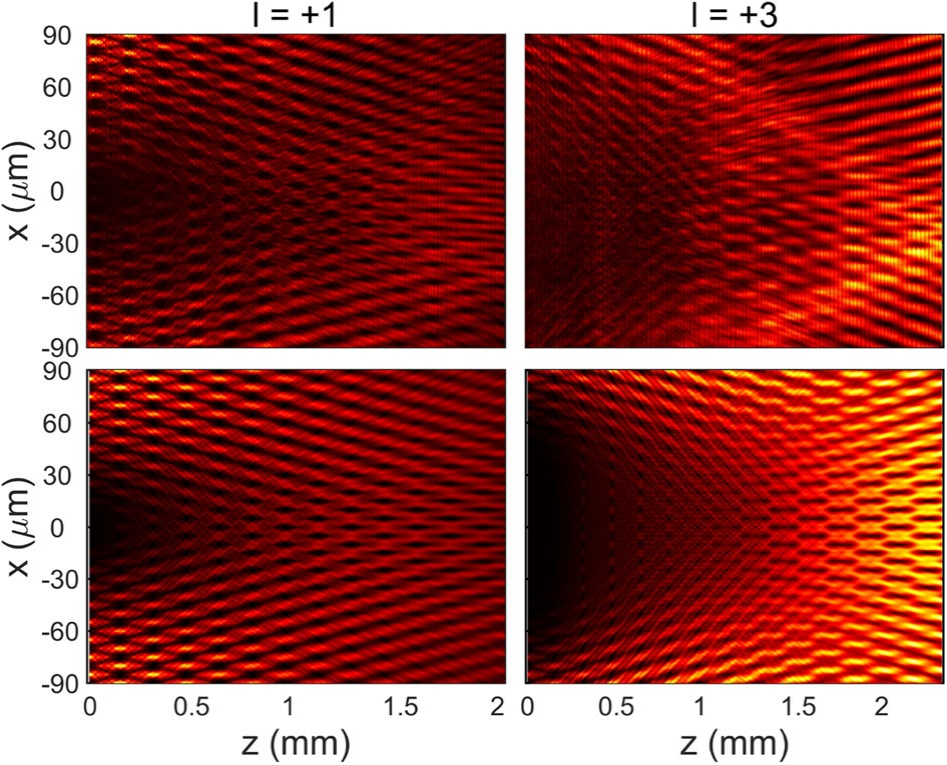
Experimental (top row) and calculated (bottom row) Talbot carpets for the incident beams with $l = +1$ (left column) and $l = +3$ (right column).

A natural (intrinsic) manifestation of the Talbot effect is a spatial dependence of the transverse near-field intensity distribution previously integrated over the second transverse coordinate^32^, which is also known as a *Talbot carpet*. It provides valuable information on the structural parameters and the light illuminating the structure. The Talbot carpets can be easily obtained for both 1D structures and plane waves. However, an obstacle arises when we refer to the optical vortices diffracted at a 2D diffraction grating. This obstacle concerns the complex spatial intensity distribution over the unit cell of a specific image, leading to the dependence of the results on the integration limits. Nevertheless, we obtained the Talbot carpets for the configuration under study. To do that, we integrated all the intensity profiles corresponding to the coordinates *z* over two rows of unit cells at the image center in the *y* direction. The results of the integration are presented in Fig. 6 for $l = +1$ and $l= +3$. These dependences have the structures well-ordered in both the transverse and forward direction. A number of the Talbot planes can be seen in all the cases. The calculated Talbot length ($Z_T = 2 \Lambda ^2 / \lambda$), i.e., the distance between the neighboring Talbot planes, was $316 \,\upmu \hbox {m}$, which is slightly different from a measured value of $324 \, \upmu \hbox {m}$. The numerically calculated Talbot carpets are in good agreement with the experimental ones.

## Discussion

**Figure 7:**
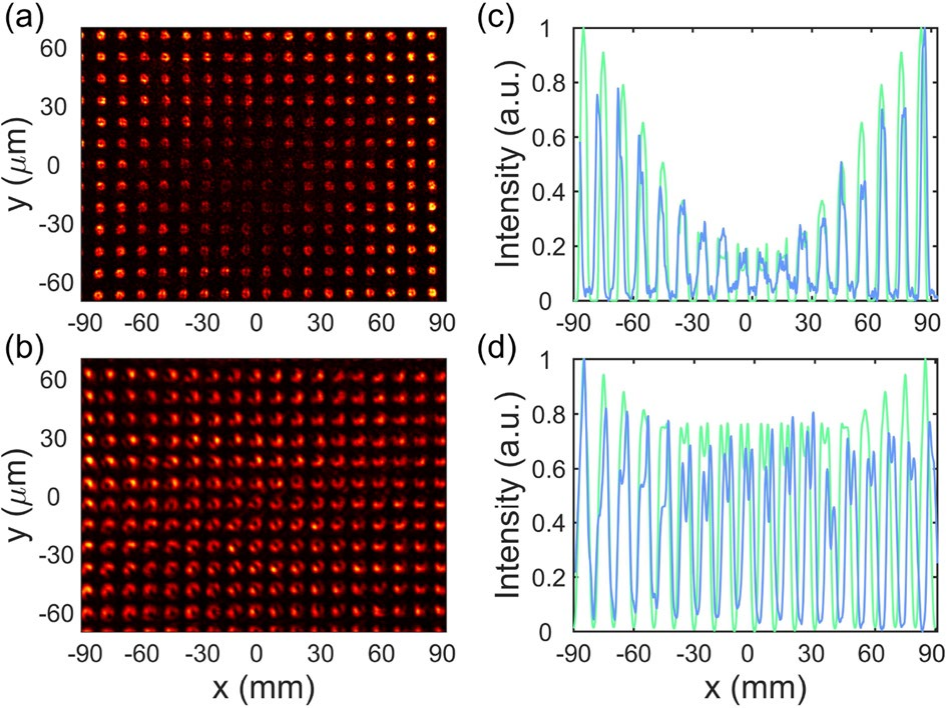
Illustration of the self-healing effect. Experimental intensity profiles for the optical vortex ($l = +1$) at (**a**) $1 Z_T$ and (**b**) $4 Z_T$ and corresponding calculated (green) and measured (blue) intensity distributions in the transverse direction. The plots in (**c**, **d**) corresponds to the plots in (**a**, **b**), respectively.

The Talbot carpets shown in Fig. 6 manifest themselves in the *self-healing* effect consisting in flattening of the central dip in the intensity distribution related to the optical vortex, i.e., restoring the image of the object plane. This effect was predicted for the optical vortices in^34^. It was attributed to a transverse energy flow from the peripheral high-intensity area of the annular beam to the central null intensity area. As can be seen in Fig. 7, the intensity profiles in the first Talbot planes have the low-intensity areas at the center, but become more uniform at $4 Z_T$. Under the experimental conditions, this effect takes place for a limited number of Talbot planes, specifically, for 3, 4, 5 Talbot planes at $l = +1$ and $4-7$ Talbot planes at $l = +3$. Our study showed that the self-healing effect is manifested in different ways, so the following situations are distinguished by the TC value of the incident beam: (i) if the TC is odd ($\pm 1, \pm 3, \pm 5, \ldots$), the intensity profiles in the self-healing area represent a beamlet array of ring-shaped maxima whose positions coincide with the lattice points of the grating structure or intermediate between the lattice points; (ii) if the TC is multiple of 4 ($\pm 4, \pm 8, \pm 12, \ldots$), the patterns represent a beamlet array of round spots of the almost uniform intensity, which resembles a perfect self-image of the object structure; in the next Talbot plane, the number of maxima is doubled and a half of them are located at the lattice points of the grating structure, while the others hold intermediate positions between them; (iii) if the TC is unevenly even ($\pm 2, \pm 6, \pm 10, \ldots$), the intensity patterns resemble the patterns for the previous case, but they are shifted by a half-period along any single coordinate.

Additionally, the measured intensity profiles in the Talbot planes under illumination by the optical vortices experience the angular rotation. In particular, in the case of illumination by the beams with an TC of $l = +3$, the intensity profiles taken at $4 Z_T$ are rotated clockwise by 3 deg. The change in the TC sign will result in the change in the rotation direction. This effect is only observed for the optical vortices and predicted by the calculations.

The approach used (see Sec. Methods) can be applied to the gratings of other types, including the phase ones, by specifying the grating transmission function $T(x_0,y_0)$ entering Eq. (3).

## Conclusions

In summary, we experimentally and theoretically studied the near-field diffraction of the optical vortices at a two-dimensional amplitude grating. The Talbot effect for the optical vortices in the visible range was experimentally observed and the corresponding Talbot carpets for the optical vortices were observed for the first time. It was shown that the complex field distributions in the Talbot planes have the phase singularities (optical vortices), whereas the intensity surrounding these singularities has an annular structure. The total TC of these singularities within the object area in the integer and fractional Talbot planes is retained and equal to the TC of the incident beam. It was numerically shown that the phase distributions within the unit cells of a specific image at the integer Talbot planes causes a complex structure of the bounded phase singularities of the opposite signs, i.e., the vortices and anti-vortices, so the resulting TC is zero. It was shown that the spatial configuration of the light field is a complex three-dimensional lattice of the beamlet-like optical vortices. A unit cell of the lattice of the OVs was reconstructed from experimental data and spatial evolution of the beamlet intensity and phase singularities of optical vortices was demonstrated. In addition, we observed the self-healing effect for the optical vortices, which consists in flattening of the central dip in the annular intensity distribution, i.e., restoring the predicted image of the object plane. The calculated results agree well with the experimental ones. The results obtained can be used to create and optimize the 3D OV lattices for a wide range of application areas.

## Methods

### Theoretical model

We consider the near-field diffraction of a Gaussian laser beam carrying the TC. The complex field amplitude of the optical vortex at the entrance of the grating can be written in the Cartesian coordinates $(x',y')$ as

2 $$\begin{aligned} E(x', y') = E_{0} \left( \frac{x'+isy'}{w}\right) ^{|l|}\exp \left( -\frac{x'^2+y'^2}{w^2}\right) , \end{aligned}$$

where $E_0$ is the maximum amplitude, *l* is the topological charge and *w* is the effective beam radius. The parameter $s = \pm 1$ corresponds to the positive and negative TC, respectively. Further, we assume that *l* is a positive integer. Then, the field amplitude at the entrance of the grating is

3 $$\begin{aligned} E(x_0,y_0)= T(x_0,y_0)E'(x',y'), \end{aligned}$$

where $T(x_0, y_0)$ is the transmission function of the grating. It is assumed that the grating is sufficiently thin, so in Eq. (3) we have $x' \approx x_0$ and $y' \approx y_0$. For a 2*D* grating, $T(x_0, y_0)$ will be a periodic function of the coordinates *x* and *y*; therefore, we have $T(x_0, y_0)=T(x_0 + m \Lambda _{x},y_0 + n \Lambda _{y})$, where $\Lambda _{x}$ and $\Lambda _{y}$ are the grating periods in the *x* and *y* directions and *m* and *n* are integers. The diffracted field amplitude in the near-field behind the grating at the distance *z* is given by the Fresnel–Kirchhoff integral^41^

4 $$\begin{aligned} E(x, y, z)=\frac{\exp (ikz)}{i\lambda z} \iint \limits _{-\infty }^{\infty } E(x_0,y_0) \exp \left[ i\frac{k}{2z}[(x-x_0)^2+(y-y_0)^2]\right] dx_0dy_0. \end{aligned}$$

Equation (4) can be presented in the form

5 $$\begin{aligned} E(x, y, z)= & {} E_{0}\frac{\exp [ikz + i(k/2z)(x^2+y^2)]}{i\lambda z w^l} \iint \limits _{-\infty }^{\infty }T(x_0,y_0) (x_0+iy_0)^l \nonumber \\&\exp \left[ -\alpha (x_0^2+y_0^2)-i\frac{k}{z}(xx_0+yy_0)\right] dx_0dy_0, \end{aligned}$$

where $\alpha = (1/w^2-ik/2z) = 1/w^2(1-ikw^2/2z)$. Since $T(x_0, y_0)$ is a periodic function of the coordinates (*x*, *y*) with the periods $\Lambda _x$ and $\Lambda _y$, then it can be expanded into the Fourier series

6 $$\begin{aligned} T(x_0, y_0)=\sum _{m,n}t_{mn}\exp [i2\pi (mG_x x_0 + nG_y y_0)], \end{aligned}$$

where $G_x = 1/\Lambda _x$ and $G_y = 1/\Lambda _y$, $t_{mn}$ are the Fourier coefficients and

7 $$\begin{aligned} t_{mn}=\frac{1}{\Lambda _x\Lambda _y} \int \limits _{-\Lambda _y/2}^{\Lambda _y/2} \int \limits _{-\Lambda _x/2}^{\Lambda _x/2} T(x_0,y_0) \exp \left[ -i2\pi (mG_xx_0 + nG_yy_0)\right] dx_0dy_0. \end{aligned}$$

Using the binomial distribution, the term $(x_0 + iy_0)^l$ in Eq. (5) can be rewritten as

8 $$\begin{aligned} (x_0+iy_0)^l = \sum _{q=0}^{l}C^{l}_{q} x_0^q(iy_0)^{l-q}, \end{aligned}$$

where $C^{l}_{q}=\frac{l!}{q!(l-q)!}$ are the binomial coefficients. Accounting for Eqs. (6) and (8), Eq. (5) takes the form

9 $$\begin{aligned} E(x, y, z)= & {} E_{0}\frac{\exp [ikz + i(k/2z)(x^2+y^2)]}{i\lambda z w^l} \sum _{m,n}t_{mn} \sum _{q=0}^{l}C^{l}_{q}(i)^{l-q} \nonumber \\&\times \iint \limits _{-\infty }^{\infty }dx_0dy_0 x_0^q\exp \left[ -\alpha x_0^2+i(2\pi mG_x-\frac{k}{z} x)x_0\right] y_0^{l-q}\exp \left[ -\alpha y_0^2+i(2\pi nG_y-\frac{k}{z} y)y_0\right] . \end{aligned}$$

The integrals in Eq. (9) can be calculated using the following reference integral^42^

10 $$\begin{aligned} \int \limits _{-\infty }^{\infty }u^m\exp (-p u^2-t u)du = \pi ^{1/2}\left( \frac{i}{2}\right) ^m p^{-(m+1)/2} \exp \left( \frac{t^2}{4p}\right) H_m\left( \frac{it}{2p^{1/2}}\right) , \end{aligned}$$

where $H_m$ are the Hermite polynomials, and the result is

11 $$\begin{aligned} E(x,y,z)= & {} \left( -\frac{1}{2}\right) ^l\frac{\pi w^2}{i\lambda z(1-ia)^{1+l/2}} \nonumber \\&\times E_{0}\exp [ikz+i(k/2z)(x^2+y^2)] \sum _{m,n}t_{mn} \sum _{q=0}^{l}C^{l}_{q}(i)^q H_q \nonumber \\&\quad \left( \frac{b_{xm}w}{2(1-ia)^{1/2}}\right) H_{l-q}\left( \frac{b_{yn}w}{2(1-ia)^{1/2}}\right) , \end{aligned}$$

Here, $b_{xm}=2\pi mG_x-(k/z)x$ and $b_{yn}=2\pi nG_y-(k/z)y$, $a=kw^2/2z$. It can be shown that

12 $$\begin{aligned} \sum _{q=0}^{l}C^{l}_{q}(i)^q H_q\left( \frac{b_{xn}w}{2(1-ia)^{1/2}}\right) H_{l-q} \left( \frac{b_{ym}w}{2(1-ia)^{1/2}}\right) = \frac{i^l w^l}{(1-ia)^{l/2}}\left( b_{xn}+ib_{ym}\right) ^l. \end{aligned}$$

Finally, taking into account Eq. (4) the diffracted field can be represented as

13 $$\begin{aligned} E(x,y,z)= & {} i^{l-1}\left( -\frac{1}{2}\right) ^l \frac{\pi w^{l+2}}{\lambda z (1-ia)^{l+1}} E_{0}\exp [ikz+i(k/2z)(x^2+y^2)] \nonumber \\&\sum _{m,n}t_{mn}(b_{xm}+ib_{yn})^l \exp \left[ -\frac{(b_{xm}^2+b_{yn}^2)w^2}{4(1-ia)}\right] . \end{aligned}$$

The intensity and phase profiles of the diffracted field in an arbitrary plane at the coordinate *z* can be calculated using Eq. (13).

### Fabrication and characterization of the sample

The sample was a transparent quartz plate, which was polished to the optical quality and then coated with a silver film with a thickness of about 200 nm. The grating was fabricated by ion etching using a focused ion beam setup (FB-2100, Hitachi). The structure represents a 2D regular array of round holes, as shown in Fig. 1b. The structure contains 40 periods along each axis and has overall sizes of $400 \times 400 \, \upmu {\hbox {m}}^2$. The respective periods were $10 \, \upmu \hbox {m}$, while the hole diameter was about $\sim 5 \, \upmu \hbox {m}$, so the duty cycles of the structure were 0.5. The use of a nontransparent silver coating allowed us to achieve the high level of the amplitude modulation of the intensity, as can be seen in the optical microscopy image presented in Fig. 1c.
